# Postnatal depression among women availing maternal health services in a rural hospital in South India

**DOI:** 10.12669/pjms.312.6702

**Published:** 2015

**Authors:** Avita Rose Johnson, Serin Edwin, Nayanthara Joachim, Geethu Mathew, Shwetha Ajay, Bobby Joseph

**Affiliations:** 1 Dr. Avita Rose Johnson, MD, DNB. Assistant Professor, Department of Community Health, St. John’s Medical College, Bangalore, India; 2 Dr. Serin Edwin, MBBS. Intern, Department of Community Health, St. John’s Medical College, Bangalore, India; 3 Dr. Nayanthara Joachim, MBBS. Intern, Department of Community Health, St. John’s Medical College, Bangalore, India; 4 Dr. Geethu Mathew, MBBS. Postgraduate student, Department of Community Health, St. John’s Medical College, Bangalore, India; 5 Dr. Shwetha Ajay, MBBS. Postgraduate student, Department of Community Health, St. John’s Medical College, Bangalore, India; 6 Dr. Bobby Joseph, MD, DNB. Professor, Department of Community Health, St. John’s Medical College, Bangalore, India

**Keywords:** Postnatal Depression, Mental Health, Pregnancy, Rural women

## Abstract

**Background and Objective:**

Postnatal depression, with an estimated prevalence of 13-19%, causes significant impairment of mental health among women worldwide and has long term consequences. However, more than half of all cases are not detected by healthcare providers. Screening for postnatal depression has not been given importance in maternal health programs in India. Our objective was to screen for postnatal depression among women attending a rural hospital in India, immediately postpartum and at 6-8 weeks post-delivery, and to study associated factors.

**Methods:**

A cross sectional study was done on 123 postnatal women attending a rural maternity hospital in Karnataka, South India, of whom 74 women were interviewed within one week of childbirth, and 49 women at 6-8 weeks post-delivery. The Edinburgh Postnatal Depression Scale was used to screen for postnatal depression.

**Results:**

About 45.5% of the women screened positive for postnatal depression (44.6% of all subjects within one week of delivery and 46.9% at 6-8 weeks after delivery). Postnatal depression was significantly associated with mood swings during antenatal period, staying with the family of birth during pregnancy and away from their husbands, and was significantly higher among women who perceived their life as stressful and having a low self-esteem (P<0.05)

**Conclusions:**

This study found a high prevalence of postnatal depression in women in rural Karnataka. This underlines the need for incorporating screening for postnatal depression in the routine care of women during pregnancy and delivery.

## INTRODUCTION

Postnatal depression, also known as postpartum depression is defined as depression with onset usually within 6 weeks of delivery.^1^ Symptoms are found to occur anytime from immediately after delivery to up to a year post delivery.^2^ Postnatal depression has been linked to lesser quality mother-child interactions,^3^ insensitive engagement of mothers with infants^4^ and even negative feelings towards the baby.^2^ This has been found to have several immediate and long term adverse effects on children. Children of depressed mothers tend to have less effective sharing and less initial sociability with strangers,^5^ more behavioral problems,^6^ more instances of malnutrition,^7^ as well as significantly affected cognitive and emotional development in the long run.^8^,^9^ Poor marital and familial relationships, and adverse effects on the mental health of partners are also some of the known consequences of postnatal depression.^10^

Prevalence estimates range from 13 to 19% in western studies.^11^ However, in low/middle income countries, prevalence of postnatal depression has been found to be higher.^12^ Studies in India have found the prevalence of postnatal depression ranging from 11 to 26.3%.^13^-^17^ There is however, a lack of data on postnatal depression among women in rural Karnataka, a state in the south of India.

Despite the adverse consequences, it has been found that more than half the cases of postnatal depression are not detected by healthcare providers.^18^ This scenario calls for more studies on postnatal depression, in an attempt to better understand the disease and its associations, with a view to prevention, early diagnosis and management, especially in rural areas of India. Screening tools and the acquisition of data on high-risk factors would aid in early recognition and management of postnatal depression.^19^ This is especially crucial in low and middle income regions like India, which have high rates of postnatal depression. Such resource-poor societies could therefore benefit from more studies on the contributing factors at play in the development of this disease.

The aim of this study was to screen and detect postnatal depression among women attending a rural hospital in Karnataka, South India, at the immediate postpartum period and at 6-8 weeks post-delivery, and to study the associated risk factors.

## METHODS

Subjects were recruited from women attending a rural maternity hospital in Ramnagara District of Karnataka. Based on a previous study,^17^ the sample size was calculated to be 121. The study population consisted of two groups of patients: the first group included women admitted in the post natal ward immediately after delivery; and the second group comprised those visiting the out-patient service for immunization of their infants at 6-8 weeks post childbirth. Consecutive sampling was used for the recruitment. Exclusion criteria were inability to give consent or inability to comprehend the questions of the study. One hundred twenty five patients were approached; two patients, both being unable to comprehend the questions of the study, were excluded in accordance with the above criteria. A total of 123 subjects were thus included in the study, 74 belonging to the first group of women in the immediate postnatal period, and 49 at 6-8 weeks post-delivery. The two groups of women were distinct and the second group was not a subset of the first. The study period was from November 2012 to January 2013.

After obtaining written informed consent, the participants were administered the study questionnaire, in a separate room to ensure privacy. The questionnaire consisted of the following: socio-demographic details and medical history, such as age, education, occupation, religion and income, history of psychiatric disorders, obstetric history including history of abortions and child death, details of present pregnancy and delivery, sex of the child, mode of delivery, as well as social factors, such as alcoholism in the husband and familial pressure to have a male child. *Edinburgh Postnatal Depression Scale (EPDS)* was then administered, which is a 10 item scale that has been widely used in screening for Postnatal Depression, including in the Indian setting.^7^,^15^ It has been found to have an overall reliability (Cronbach’s alpha) of 0.79,^20^ sensitivity of 86% and a specificity of 78%.^21^ The scale was translated and back-translated into the local language (Kannada) for our study. Subjects who scored 13 or above in EPDS were considered to have screened positive for postnatal depression and were referred to a senior physician for further evaluation and management.

Data was analyzed using SPSS Version 18.0. Descriptive statistics were used to study the population in terms of frequency distributions for socio-demographic characteristics and details of antenatal care and delivery. EPDS scores were described in terms of means and standard deviations. T tests/Chi square tests were used to compare groups, in terms of demographic details, details of pregnancy, delivery, history of psychiatric illnesses, social factors, and time since delivery.

## RESULTS

Most of the subjects in our study population were between 20-25 years, were school or high-school educated and were housewives. Majority belonged to the Hindu religion (Table-I). Mean age at marriage was 20.11± 2.47. Mean per capita family income was Rs.1978.24 ±2744.96 per month. Over 80% of women in both groups had had their first ante-natal checkup within three months of pregnancy. 28.5% were multiparous. Most of the subjects had delivered the present child via normal delivery(78.9%). 18.7% had delivered by caesarean section. There was no statistically significant difference in the socio-demographic characteristics of the women in the two groups, that is, those who were interviewed within one week post-delivery and those at 6-8 weeks post-delivery.

**Table-I:** Socio-demographic profile of the study subjects.

	<1 week post delivery No. (%) n=74	At 6- 8weeks post delivery No. (%) n=49	
Age Group (years):			
<20	7(9.5)	5(10.2)	P = 0.646
20-25	56(75.7)	36(73.5)	
26-30	9(12.2)	6(12.2)	
>30	2(2.7)	2(4.1)	
Education:				
Uneducated	6(8.1)	2(4.1)	P = 0.517
Secondary/High school	8(10.8)	15(30.6)	
10th standard	22(29.7)	10(20.4)	
Pre-university	26(35.1)	15(30.6)	
Degree	12(16.2)	7(14.3)	
Occupation:				
Unemployed(Housewife)	63 (85.1)	44 (89.8)	P = 0.771
Unskilled	7 (9.5)	0 (0)	
Skilled	2 (2.7)	3 (6.1)	
Semi-Profession	2 (2.7)	2 (4.1)	
Religion:				
Hindu	69 (93.2)	48 (98.0)	P=0.193
Muslim	4 (5.4)	1 (2.0)	
Christian	1 (1.4)	0 (0)	
Type of Family:				
Joint	35(47.3)	15(30.6)	P=0.375
Nuclear	11(14.9)	12(24.5)	
Three generation	28(37.8)	22(44.9)	

### EPDS scores

Screening by EPDS gives a maximum score of 30 and women who scored more than 13 are considered screened positive for postnatal depression. Mean score for women in the immediate postnatal period was 11.27±3.92 and that for those at 6-8 weeks post-delivery was 11.86±4.35. There was no difference between these two means (Table-II). 44.6% among women within one week of delivery and 46.9% among women within 6-8 weeks of delivery screened positive for postnatal depression. There was no significant difference in the proportion of women who screened positive for postnatal depression at less than one week and at 6-8 weeks post-delivery. Postnatal depression was found to be significantly associated with mood swings during pregnancy (P=0.027, Table-III). Women who did not live with their husbands around the period of delivery (that is, they resided with their family of birth) were found to be more likely to have postnatal depression (P=0.010). Women who had postnatal depression were found to be more likely to perceive their life as stressful (P=0.011), as well as to have low self-esteem (P=0.041).

**Table-II:** Postnatal depression and Mean EPDS scores among the 2 groups of women.

	N	Screen Positive by EPDS			Mean EDPS score	SD
		Yes (%)	No (%)				
Time since delivery:							
< 1 week	74	33(44.6)	41(55.4)		11.27	3.92	P = 0.33
6-8 weeks	49	23(46.9)	26(53.1)	P= 0.789	11.86	4.35		
Total	123	56(45.5)	67(54.5)		11.50	4.09		

**Table-III:** Associations of postnatal depression.

	Screen Positive by EPDS	
	Yes(%)	No (%)
Moods swings during antenatal period			
Yes	11(73.3)	4(26.7)	P = 0.027*
No	45(41.7)	63(58.3)	
Residence during Delivery				
Family of Birth	16 (69.6)	7 (30.4)	P=0.010*
With Husband	40 (40.0)	60 (60.0)	
Perception of one’s lifeas stressful				
Yes	14 (73.7)	5 (26.3)	P=0.011*
No	42 (40.4)	62 (59.6)	
Low Self Esteem				
Yes	18 (62.1)	11 (37.9)	P=0.041
No	38 (40.4)	56 (59.6)	

No significant association was found between screening positive for postnatal depression and age, education, occupation, religion, type of family, family history of psychiatric illness, complications during pregnancy and childbirth. None of the 123 women reported a history of previous psychiatric illness or had undergone treatment for a psychiatric illness.

## DISCUSSION

Mental health is a significant decisive factor in the well-being of any community. Depression in particular, has been receiving increased attention in recent years, contributing to substantial disease burden throughout the world. About 350 million people live with depression in the world.^22^ Strategies to alleviate its impact, therefore, are of utmost importance. Pregnancy and childbirth, in particular, are times of great physical and emotional stress for women. Mental well-being during this time is therefore of added importance. Postnatal depression is a disease that can cause serious impairment to this well-being. Postnatal depression poses a risk for the mother child relationship and infant development outcomes. Postnatal depression among mothers can have a deleterious effect in cognitive as well as emotional development of the infants.^23^ It has been seen that treatment of postnatally depressed mothers results in improving the quality of the mother–infant interaction and relationship. Early recognition and management of postpartum depression can help ensure better outcomes for the mother and child.^24^

The present study found that 44.6% of those in the immediate postnatal period and 46.9% of those at 6-8 weeks post-delivery screened positive for postnatal depression. These findings are in agreement with previous studies that have found a high prevalence of postnatal depression, of up to 42%, in resource-poor countries.^13^,^25^,^26^ These consistently high rates of postnatal depression found in low-income countries, and its serious associations underline the need for the incorporation of postnatal depression screening tools in the routine management of mothers in the postnatal period. In a cross sectional community based study done in Bangladesh the prevalence of post partum depression at 6 to 8 weeks was 22% which is lower compared to our study. Possible reasons could be that hospital based studies may result in the overestimation of the true prevalence of postnatal depression compared to community based studies. The healthy mother and baby may not turn up to the clinics after 6 weeks leading to a low prevalence of depression in community based studies.^27^

In a cross-sectional hospital based study done at Udupi, Karnataka, the prevalence of postpartum depression was noted to be 11.3% within one week and 15.8% at 6-8 weeks.^13^ A similar trend of slightly more instances of depression later in the postnatal period as compared to the immediate postnatal period was seen in the present study as well, with 46.9% of women at 6-8 weeks post-delivery screening positive for postnatal depression compared to 44.6% of those in the immediate postnatal period. Though this difference was not statistically significant it could be explained considering that in the immediate postnatal period, the symptoms of depressive illness may be alleviated by the positive feelings associated with the recent birth of a child and the increased social support typically associated with childbirth in the Indian society. Increased exposure to healthcare personnel around this period could also have played a role. The present study showed a much higher prevalence of postpartum depression than the Udupi study. This may be due to the difference between the two districts in terms of poverty levels, education, employment opportunities and overall health care, all of which are known risk factors for post partum depression.^28^,^29^ Another reason could be that, unlike the Udupi study, the present study included women with perinatal complications and chronic diseases, who are a high risk group for postpartum depression.^30^

Risk factors for postnatal depression as detected by previous studies in the Indian society include lower socioeconomic status, multiparity, disappointment with the sex of the child, female sex of the child and congenital malformations of the child.^6^,^13^ Postnatal depression has also been found to be associated with relationship difficulties with the mother-in-law and parents, adverse life events during pregnancy, lack of physical help,^14^ husband’s use of alcohol, education less than 5 years and a family history of depression.^21^ The present study did not find any associations between postnatal depression and adverse socio-cultural factors like pressure to have a male child, unlike in another study in Tamil Nadu.^14^ This could have been due to the higher level of education among our study population.

We found that residing with the family of birth during pregnancy as opposed to with the husband, was related to postnatal depression. In rural India, it is a common practice for women to stay with their own family of birth during the perinatal period. Our findings suggest that this could be a factor contributing to postnatal depression. It may be possible that staying with the husband during the time around delivery, may result in the woman perceiving more support from the husband, protecting against postnatal depression.

Though we did not find any associations with other social factors, it was noted by the investigators that the subjects showed a tendency to shy away from admitting to adverse social factors, especially alcohol addiction in the husband. This may not have been the actuality. This observation was similar to that by investigators in a previous study in Tamil Nadu,^14^ who noted that the fear of societal rebuke and cultural dictums that expected women in India to respect their husbands despite all else, prevented women from being open about their relationships. We also found depressed women to be more likely to report stress and have low self-esteem. A similar finding was evinced in other studies, where low self-esteem and stress were found to be predictors of postpartum depression.^31^-^32^

Postnatal depression was found to be significantly associated with mood-swings in the antenatal period in the present study. This is in accordance with previous findings suggesting depression during pregnancy as a strong predictor for postnatal depression.^6^,^14^,^33^,^34^ This implies that it would be beneficial to have a low threshold of suspicion for depressive symptoms in the postnatal period in such patients.

This study complements previous studies that found a high prevalence of postnatal depression in less developed regions of the world. In this scenario, it is important that screening for postnatal depression using tools such as the Edinburgh Postnatal Depression Scale be incorporated in the routine care of women during pregnancy and delivery. Psychosocial interventions could be initiated for mothers who screen positive under maternal welfare programmes. In addition, according to our findings, women who report mood swings during the antenatal period are at possible risk for the development of postnatal depression. Anticipatory guidance and intensive care could be provided for such patients by obstetricians, midwives or public health nurses. Further studies could explore if interventions such as counseling and follow up could prevent postnatal depression in such patients. Patients who live away from their husband’s during pregnancy also seem to be at a higher risk of developing postnatal depression. The socio-cultural factors that are responsible for this finding need to be evaluated further in future studies.

### Limitations

Practical constraints restricted this to a hospital based assessment. A community based study with a large sample size could contribute to a better understanding of possible social and environmental factors contributing towards the development of postnatal depression. As this was a cross-sectional study, no inference regarding causation could be made. A validated tool was not used for assessing self-esteem and stress in the study subjects.
